# Vascular Abnormalities and the Role of Vascular Endothelial Growth Factor in the Epileptic Brain

**DOI:** 10.3389/fphar.2020.00020

**Published:** 2020-02-04

**Authors:** Ari Ogaki, Yuji Ikegaya, Ryuta Koyama

**Affiliations:** ^1^ Laboratory of Chemical Pharmacology, Graduate School of Pharmaceutical Sciences, The University of Tokyo, Bunkyo, Japan; ^2^ Center for Information and Neural Networks, National Institute of Information and Communications and Technology, Suita City, Japan

**Keywords:** antiepileptic drug, neurovascular abnormalities, astrocyte, pericyte, endothelial cell, blood–brain barrier

## Abstract

Epilepsy is a chronic neurological disorder generally defined to be caused by excessive neuronal activity. Thus, excessive neuronal activity is the main target of the currently used antiepileptic drugs (AEDs). However, as many as 30% of epileptic patients show drug resistance to currently available AEDs, which suggests that epilepsy should be attributed not only to neuronal cells but also to other brain cells, such as glial cells and vascular cells. Astrocytes, pericytes, and endothelial cells in particular comprise the blood–brain barrier (BBB), which tightly regulates the exchange of substances between the brain parenchyma and the circulating blood. It has been proposed that BBB dysfunction, especially barrier leakage, exacerbates epileptic progression, and conversely, that epileptic seizures induce barrier leakage. Furthermore, several studies have shown that BBB dysfunction is one of the main causes of drug resistance in epilepsy. To better understand the mechanisms that link BBB dysfunction and intractable epilepsy to gain insights for the future development of treatments, we review and discuss the relationships between epilepsy and brain vascular abnormalities, mainly by focusing on vascular malformation, BBB dysfunction, and excessive angiogenesis. Because these abnormalities have been reported to be caused by vascular endothelial growth factor (VEGF) in the ischemic brain, we discuss the possible role of VEGF in vascular abnormalities in the epileptic brain, in which the upregulation of VEGF levels has been reported. Both glial cells and endothelial cells express VEGF receptors (VEGFRs); thus, these cells are likely affected by increases in VEGF during seizures, which in turn could cause vascular abnormalities. In this review, we review the possible role of VEGF in epilepsy and discuss the mechanisms that link vascular abnormalities and intractable epilepsy.

## Introduction

Epilepsy is a chronic neurological disorder accompanied by spontaneous and frequent seizures, which are induced by the aberrant hyperactivity of neurons. The main effect of existing antiepileptic drugs (AEDs) is the prevention of the excessive activity of neurons, mainly by activating ion channels expressed in neurons; however, as many as 36% of epileptic patients show drug resistance (Kwan and Brodie, 2000). Drug-resistant epilepsy is defined as the “failure of adequate trials of two tolerated and appropriately chosen and used AED schedules (whether as monotherapies or in combination) to achieve sustained seizure freedom” (Kwan et al., 2010). Temporal lobe epilepsy (TLE) is a common drug-resistant epilepsy, and surgical treatments are often used for the treatment of TLE (Jallon et al., 2001). Therefore, new drug targets are needed.

**Figure 1:**
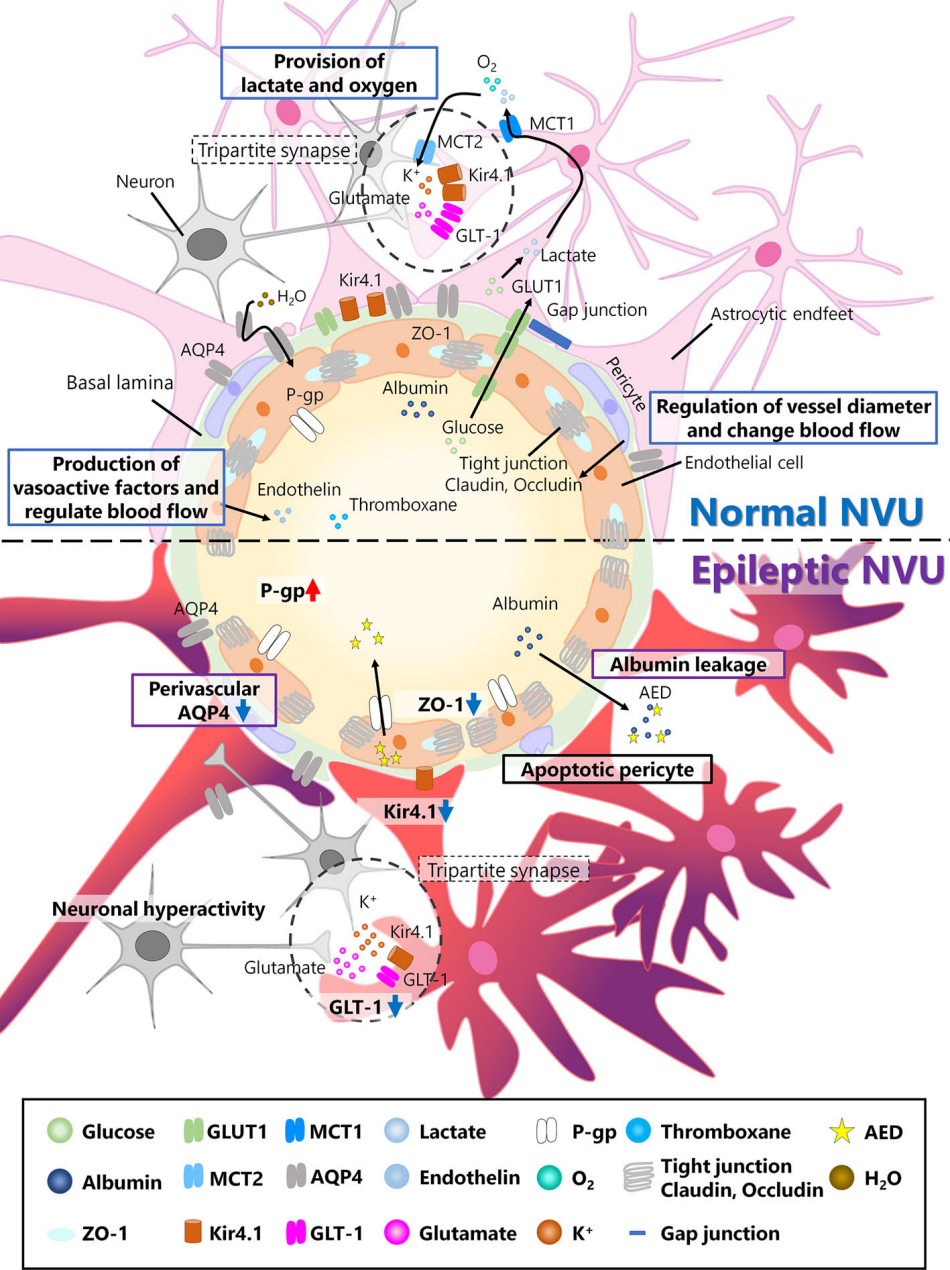
Neurovascular unit (NVU) in the normal and epileptic brain. The NVU is comprised of neurons, astrocytes, pericytes, and endothelial cells. The basal lamina also exists between endothelial cells and astrocytes and is covered with astrocytic endfeet in the normal NVU. In the normal NVU, pericytes regulate the vessel diameter and change in blood flow. Endothelial cells are connected by tight junction proteins such as claudin and occludin, and ZO-1 is a type of occludin. Glucose is transported from the blood flow to astrocytes via glucose transporter type 1 (Glut1), metabolized into lactate, and discharged into extracellular space via monocarboxylate transporter 1 (MCT1). This lactate is transported to neurons via MCT2. Additionally, oxygen is also provided to neurons by astrocytes. Astrocytes and pre/post synapses comprise tripartite synapses. At tripartite synapses, potassium ions and glutamate released from neurons flow into astrocytes via inwardly rectifying potassium (Kir) 4.1 channels or glutamate transporter 1 (GLT-1), both of which are expressed around astrocytic endfeet. Additionally, aquaporin (AQP) 4, which is also expressed in astrocytes, regulates water transport. Endothelial cells produce vasoactive factors, such as thromboxane and endothelin, and regulate blood flow. In epileptic NVUs, ZO-1 expression is downregulated, leading to the apoptosis of pericytes, which induces blood–brain barrier (BBB) leakage. Albumin released from the blood flow into the brain parenchyma in the epileptic brain can be a cause of drug resistance. Kir 4.1 and GLT-1 are downregulated in astrocytes, which fail to regulate ion and neurotransmitter homeostasis. Perivascular AQP4 downregulation induces an imbalance in water influx. P-gp, which is expressed in endothelial cells and is associated with antiepileptic drug (AED) excretion, is upregulated in the epileptic NVU.

Approximately 20 years ago, the concept of the neurovascular unit (NVU) was proposed (Harder et al., 2002). According to this concept, neuronal activity is regulated not only by neurons themselves but also by other cells surrounding blood vessels, including glial cells, pericytes, and endothelial cells (**Figure 1**). Glial cells, especially astrocytes, provide lactate and oxygen to support neuronal health. Pericytes have actin–myosin systems that are associated with cell contraction, which enables the regulation of vessel diameters and changes in blood flow that could affect neuronal activity. Endothelial cells produce vasoactive factors to regulate vessel diameters, possibly resulting in changes in neuronal activity (Muoio et al., 2014). These changes in the brain microenvironment together could affect neuronal activity and brain function.

**Figure 2:**
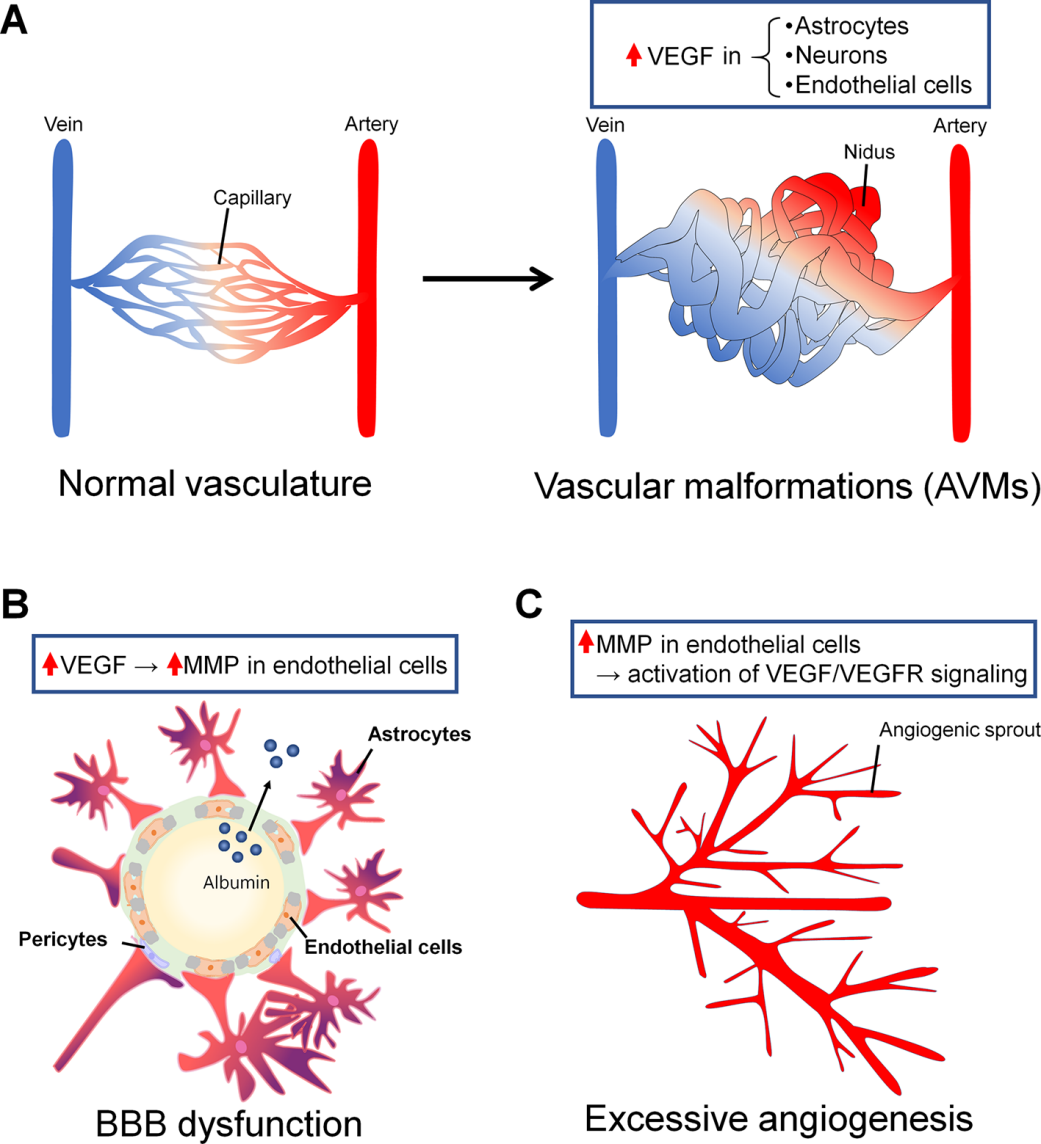
Vascular abnormalities in epilepsy associated with vascular endothelial growth factor (VEGF). **(A)** Arteriovenous malformations (AVMs) are one of the major vascular malformations found in the epileptic brain. In normal vasculature, arteries and veins are connected by capillaries (right). In AVMs, there are some regions in which arteries and veins are not connected by capillaries and are instead directly connected by blood tumors called niduses (left). In astrocytes, neurons, and endothelial cells around and in niduses, VEGF expression is increased. **(B)** BBB dysfunction can be a cause or a consequence of epilepsy, or it can be both a cause and a consequence at the same time. BBB dysfunction induces albumin leakage and resistance to antiepileptic drugs (AEDs). VEGF induces an increase in MMP levels in endothelial cells, leading to BBB leakage. Albumin leaked from the blood flow binds AEDs and promotes drug resistance. **(C)** Angiogenesis is associated with BBB dysfunction in the epileptic brain. Neo vessels sprout from the pre-existing vessels. Matrix metalloproteinase (MMP) overexpression in endothelial cells could induce increases in VEGF/VEGFR signaling levels and angiogenesis.

In this review, in considering possible new therapeutic targets for epilepsy, we mainly focus on the NVU, which is comprised of glial cells, pericytes, and endothelial cells. In the epileptic brain, several studies have suggested that the NVU exhibits abnormal characteristics. Although the topic on vascular structure abnormalities in epilepsy has been reviewed elsewhere (Morin-Brureau et al., 2012; Marchi and Lerner-Natoli, 2013; Josephson et al., 2015), here we specially focused on vascular malformation, blood–brain barrier (BBB) dysfunction, and excessive angiogenesis (**Figure 2**). In a brain with vascular malformation, vessel aggregations, for example, compress the brain parenchyma, resulting in abnormalities in the layer structures of neurons in the cerebral cortex. The malformation of neuronal layers could induce the hyperactivity of neurons, which results in epilepsy. In a brain with BBB dysfunction, albumin leaked from cells in the blood is taken up by astrocytes and attenuates the adjustment of extracellular ion concentrations by astrocytes, possibly resulting in neuronal hyperactivity. Additionally, BBB leakage could induce excessive angiogenesis and increase blood flow. These abnormalities are possibly induced by vascular endothelial growth factor (VEGF), whose expression is reported to be increased in the brains of patients with TLE (Rigau et al., 2007; Castañeda-Cabral et al., 2019). VEGF, which is produced and secreted from several types of cells, such as fibrocytes, myocytes, and astrocytes, under pathological conditions (de Vries et al., 1992), modulates the function of several types of cells, especially endothelial cells (Lee et al., 2007). VEGF induces the proliferation and migration of endothelial cells, which results in the promotion of angiogenesis (Hoeben et al., 2004). Therefore, we will describe vascular abnormalities in the epileptic brain, mainly by focusing on the roles of VEGF in their development.

## Vascular Malformation

It is commonly known that epileptic seizures are often associated with vascular malformations (Stefan and Hammen, 2004), and vascular malformations themselves can be a cause of epilepsy in some cases. Intracranial vascular malformations, which have been suggested to be a cause of epilepsy, are divided into two major types: arteriovenous malformations (AVMs) and cerebral cavernous malformations (CCMs) (Brown et al., 2005). These malformations often develop in the fetus between 3 and 8 weeks and are induced by the abnormal differentiation of the mesoderm. In AVMs, abnormal structures called “niduses” are found, in which arteries and veins are connected with blood flow in tumors without capillaries in the brain parenchyma (**Figure 2A**). In CCMs, aggregated venous blood vessels are observed in the brain, and these aggregations are often expanded and accompanied by slight hemorrhaging. AVMs and CCMs are common causes of intracerebral hemorrhage (ICH) in young adults; therefore, ICH itself could result in the induction of epileptic seizures. However, even AVMs and CCMs without symptomatic ICH can induce epileptic seizures. Eight percent of patients with unruptured AVMs were found to exhibit their first seizures within 5 years after the first detection of unruptured AVMs, and 58% of individuals with seizures develop epilepsy within 5 years (Josephson et al., 2011). In individuals with CCMs, there have been few studies describing the risk of seizures or epilepsy development mainly because it is difficult to detect CCM; however, one study reported that the risk of occurrence of the first seizure is 4% within 5 years after the first detection of CCMs and that the risk of development of epilepsy is 94% in CCM patients (Josephson et al., 2011). It has also been reported that patients with CCMs more likely develop epilepsy than patients with other massive lesion diseases (Lv et al., 2010).

The pathological mechanisms of epilepsy induced by AVMs and CCMs remain largely unclear. AVMs that are associated with seizures exist in various brain regions, but they are generally found in the temporal lobe (Josephson et al., 2011), and AVMs cause epileptic seizures through ischemia and impairment of the perinidal vascular reserve (Fierstra et al., 2011). One of the remarkable mechanisms that links the induction of epileptic seizures and CCMs is the deposition of hemosiderin (Leone et al., 2011). Hemosiderin, which is a pigment that includes iron derived from hemoglobin leaked from endothelial cell junctions, is rarely deposited in the healthy brain. Around deposits of hemosiderin, gliosis is often promoted, which may contribute to epileptogenesis. Thus, it is possible that AVM- and CCM-associated hemorrhage induces the deposition of hemosiderin, which includes iron, and iron could greatly contribute to the formation of focal epilepsy (Zhang et al., 2014; Josephson et al., 2015). Iron deposition causes lipid peroxidation and the alternation of protein function and could induce the disruption of the neuronal excitatory versus inhibitory balance, likely *via* disrupting the intracellular and extracellular homeostasis of neurotransmitters.

It remains unclear how AVMs and CCMs develop, but it is likely that excessive angiogenesis underlies the formation of both AVMs and CCMs (Leblanc et al., 2009). In addition, it has been suggested that the formation and progression of AVMs and CCMs are induced by the local overexpression of VEGF (Li et al., 2018; Park and Park, 2016). VEGF, which is widely recognized as the main factor that induces angiogenesis, is locally overexpressed in and around AVMs in the brain (Koizumi et al., 2002). Specifically, VEGF-C and -D and the VEGF receptors (VEGFRs) Flt-1 and -4 are overexpressed in and around niduses. In contrast, the VEGF concentration in the plasma of AVM patients was lower than the control level, and it recovered to the control level after the treatment of AVMs (Kim et al., 2008). The reason why the VEGF concentration in plasma in AVM patients is lower while the concentration in and around niduses is higher than control levels remains unknown. In AVMs, VEGF overexpression is found in astrocytes, neurons, and endothelial cells; therefore, it is possible that these cells secrete VEGF to support angiogenesis and the formation of AVMs (Li et al., 2018), although direct evidence that supports this hypothesis has not been reported.

The concentration of plasma VEGF in CCM patients is higher than the control level, and it is decreased to the control level after CCM treatment (Park and Park, 2016). The expression levels of VEGF and VEGFR in CCMs are also higher than the control levels (Rothbart et al., 1996; Uranishi et al., 2001). Furthermore, when *CCM3*, one of the genes responsible for CCM, is deleted in mice, normal VEGFR2 signaling, which is necessary for angiogenesis, is attenuated (He et al., 2010). Thus, it is possible that the mutation of *CCM3* destabilizes the VEGFR signaling pathway, inducing excessive angiogenesis and the formation of CCMs.

## BBB Dysfunction

The BBB in the brain capillary consists of endothelial cells, basement membrane, pericytes, and astrocytes. In the BBB, tight junctions formed by endothelial cells regulate paracellular flux, and the basement membrane, which is comprised of extracellular matrix secreted by endothelial cells and pericytes, is associated with vascular signaling; in addition, pericytes regulate blood flow and the infiltration of immune cells. Finally, astrocytes, which express the water channel aquaporin 4 (AQP4), are crucial for water homeostasis in the central nervous system (CNS; Daneman and Prat, 2015). The main function of the BBB is the regulation of the substances that are allowed to enter the brain parenchyma from the systemic blood flow.

The relationship between BBB dysfunction, especially BBB leakage, and epilepsy have long been studied (Oby and Janigro, 2006). BBB dysfunction has been found both in the brains of patients with epilepsy and in the corresponding animal models. In brains that undergo status epilepticus (SE), it was reported that albumin leakage was found surrounding all vessels in the hippocampus and cortex (van Vliet et al., 2007). In the hippocampus of TLE patients, albumin was also found in neurons and astrocytes that exist around vessels (van Vliet et al., 2007). The causal relationship between epileptic seizures and BBB leakage is consistent with findings from animal models of epilepsy. When rats were injected with pilocarpine to induce SE, BBB leakage was found in the hippocampal CA3 region and the dentate gyrus (Marchi et al., 2007). Furthermore, when rats were injected with kainic acid (KA) to induce SE, erythrocyte leakage in the piriform cortex and amygdala induced by SE was attenuated when rats were treated with an inhibitor of mammalian target of rapamycin (mTOR), rapamycin, which has been experimentally shown to exert anticonvulsant effects (van Vliet et al., 2016). Additionally, when BBB leakage was artificially induced by administrating hypertonic mannitol into rats, the number of seizures was increased in electric stimuli-induced SE rats (van Vliet et al., 2007).

To date, it is still debatable whether BBB dysfunction is a consequence or a cause of epilepsy. Neurons have been shown to exhibit epileptiform discharges induced by serum that is leaked from the blood flow (Seiffert et al., 2004). Furthermore, several mechanisms have been suggested that explain the leakage of thrombin and albumin from plasma into the brain parenchyma (Lee et al., 1997; van Vliet et al., 2007). It has also been suggested that BBB dysfunction triggers drug resistance in epilepsy. BBB leakage causes albumin to flow into the brain parenchyma from the plasma. Albumin decreases the concentrations of nonbinding AEDs (Marchi et al., 2009), likely decreasing the medical efficacy in terms of pharmacokinetics. Furthermore, drug excretion from the brain may be enhanced, as the overexpression of P-gp protein, which is one of the ABC transporters (ATP-binding cassette transporters) associated with drug excretion, was found to be induced after seizures (Zhu and Liu, 2004).

Several molecules, such as matrix metalloproteinase (MMP), have been suggested to induce BBB dysfunction in the epileptic brain. MMP is known to cleave extracellular matrix and degrade tight junction proteins (Lischper et al., 2010). In the cortex and hippocampus of patients with intractable epilepsy due to focal cortical dysplasia, MMP upregulation was reported (Konopka et al., 2013). In particular, the prominent upregulation of MMP-9, one of the MMP family members that is expressed in brain capillaries, was observed. In the brains of pilocarpine-induced SE rats, the increased release of glutamate by neurons increased MMP levels, leading to BBB dysfunction (Rempe et al., 2018). VEGF is another molecule associated with BBB dysfunction in the brain (Croll et al., 2004; Rigau et al., 2007). VEGF administration induced BBB leakage in rodents, and there was a positive correlation between VEGF-induced leakage and the increased activity of MMP-9 in the ischemic brain (Valable et al., 2005; Jiang et al., 2014). It remains unclear whether and how VEGF-induced BBB leakage and upregulated MMP-9 activity synergistically affect the epileptic brain.

Other molecules suggested to be associated with BBB dysfunction include platelet-derived growth factor (PDGF) and its beta receptor (PDGFRβ). PDGF is secreted from platelet and macrophages, whereas PDGFRβ is expressed in endothelial cells. PDGFRβ overexpression in endothelial cells induced the development of pericyte-microglia scar in the epileptic brain (Milesi et al., 2014; Klement et al., 2018; Klement et al., 2019). Because pericytes are essential components of BBB, pericyte-microglial scar would result in BBB leakage. Moreover, when seizure-like activities were induced in organotypic hippocampal slice cultures, the treatment of the cultures with imatinib, an inhibitor of PDGFRβ, decreased the reactivity between PDGFRβ-positive cells and capillaries which was triggered by seizure-like activities (Klement et al., 2019).

## Excessive Angiogenesis

Angiogenesis, the process of new vessel formation from pre-existing vessels, can be observed under both physiological, i.e., developmental, and pathological conditions. The density of vessels in the hippocampi from TLE patients was approximately two times higher than that in hippocampi from nonepileptic controls (Rigau et al., 2007). Moreover, the frequency of seizures was correlated with the density of the vessels in the hippocampi of TLE patients; the density of vessels in patients who had seizures less than once a month was significantly lower than that in patients who had seizures 1~20 times a month. It should also be noted that the existence of hippocampal sclerosis (HS) did not affect the density of vessels in the hippocampi of TLE patients. The increase in the density of brain vessels has been reproduced in animal models of TLE. The vessel density in the hippocampus of lithium-pilocarpine-induced SE rats was increased compared to that of the control group, especially in the chronic phase (Rigau et al., 2007). By using rat hippocampal slices cultured with KA to induce seizure-like events (SLE), it was found that the vessel density and the number of vessel branches were increased at 24 h after KA application, and these phenomena were suppressed by inhibiting neuronal activity with tetrodotoxin (Morin-Brureau et al., 2011).

Several studies have suggested that angiogenesis in the epileptic brain is correlated with VEGF-associated BBB dysfunction (Rigau et al., 2007; Morin-Brureau et al., 2012). VEGF and VEGFR2 mRNA were upregulated, and ZO-1, which is a tight junction-related protein, was down-regulated in KA-treated slice cultures (Morin-Brureau et al., 2011). Treatment with anti-VEGF antibodies attenuated the increases in vessel density and branch numbers and the decrease in ZO-1 expression. The authors also found that VEGF/VEGFR2 signaling likely induces angiogenesis and BBB dysfunction through the proto-oncogene tyrosine protein kinase Src pathway.

Endothelial cells express VEGFR, and several reports have suggested that MMP activates the VEGF/VEGFR signaling pathway in angiogenesis under pathological conditions (Ferrara, 1995; Bergers et al., 2000; Hollborn et al., 2007). However, whether and how these systems contribute to angiogenesis in the epileptic brain remain to be studied. It is possible that MMP upregulation induces an increase in VEGF, leading to angiogenesis in the epileptic brain. Even in cultured normal tissues, in which pancreatic islets and endothelial cells were cocultured, that were treated with MMP, an increase in the VEGF concentration in the culture medium and angiogenesis in endothelial cells were observed (Bergers et al., 2000). Additionally, when cancer model mice were treated with a VEGFR inhibitor or an MMP inhibitor, angiogenesis, which is commonly observed in animal models of cancer, was inhibited (Hollborn et al., 2007). These data suggest that angiogenesis may also be prevented by inhibiting VEGFR or MMP in the epileptic brain.

## Discussion

Mechanisms underlying synchronized bursting of neurons have been therapeutic targets for epilepsy. However, about one-third of patients with epilepsy remain resistant to AEDs, which suggests that the role of brain cells other than neurons should be also studied in the epileptic brain. NVU, which consists of multiple cell types including neurons, astrocytes, pericytes, and endothelial cells, can be an essential therapeutic target for epilepsy. In physiological conditions, each cell type that consists NVU plays multiple roles: pericytes regulate the vessel diameter and modulate blood flow; endothelial cells produce vasoactive factors and modulate blood flow; astrocytes provide lactate and oxygen to support neuronal survival and growth (**Figure 1**). Specifically, endothelial cells, being tightly adhered each other as a part of NVU, play an essential role to regulate the exchange of substances between brain and blood flow in BBB. However, in the epileptic NVU, the regulatory systems of substance exchange are frequently disrupted because the adhesion between endothelial cells is attenuated, phenomena referred to as BBB breakdown. BBB breakdown results in the release of albumin and its binding to AEDs, which is a cause of drug resistance. Additionally, BBB breakdown results in the excessive release of potassium and glutamate. To make matters worse, the astrocytic expression of inwardly rectifying potassium (Kir) 4.1 channels and glutamate transporter1 (GLT1) is downregulated in the epileptic brain, which could result in dysregulation of ion and neurotransmitter concentration in tripartite synapses.

VEGF/VEGFR signaling is essential for angiogenesis in CNS (Mancuso et al., 2008). VEGFR stimulation in endothelial cells activates phosphatidilinosytol-3-kinase (PI3K) and following Rac/Rho signaling as well as growth factor binding protein2 (GRB2), which leads to the activation of Cdc42. Both Rac/Rho and Cdc42 have been shown to be associated with cell proliferation. The activation of VEGFR also induces the activation of MAP kinase signaling, which is also related to cell proliferation. Thus, VEGF-triggered cell proliferation is considered to be the basis of CNS angiogenesis.

In the current review, we have mainly focused on the negative effects of VEGF/VEGFR signaling in the epileptic brain, including vascular malformations, BBB dysfunction, and excessive angiogenesis. However, it should be noted that VEGF/VEGFR signaling also has neurotrophic effects in CNS including the induction of neurite outgrowth and suppression of neuronal death (Rosenstein et al., 2010). Furthermore, several studies have reported that VEGF has antiepileptic effects in pilocarpine-induced SE rats (McCloskey et al., 2005; Han et al., 2017; Lenzer-Fanara et al., 2017). VEGF treatment reduced spontaneous discharges of neurons in brain slices (McCloskey et al., 2005) and promoted hippocampal neurogenesis *in vivo*, improving recognition ability (Han et al., 2017). Additionally, VEGF treatment changed the morphology of astrocytes, especially their branches (Lenzer-Fanara et al., 2017), which may affect hippocampal function after seizures because astrocytic feet are essential components of tripartite synapse (**Figure 1**).

Enhanced VEGF/VEGFR signaling may contribute to attenuating cognitive deficits after epileptic seizures by promoting adult neurogenesis in the dentate gyrus (Rosenstein et al., 2010; Han et al., 2017). The relationship between adult neurogenesis and the NVU has been studied (Goldman and Chen, 2011), and a recent study reported that microvascular hemodynamics modulate adult neurogenesis (Shen et al., 2019). Furthermore, VEGF administration suppressed neuronal loss in the hippocampal CA1 region 24 h but not one month after pilocarpine-induced SE in rats (Nicoletti et al., 2010). Additionally, when rats were treated with VEGF and angiopoietin-1, VEGF-induced vascular permeability was suppressed without affecting neuroprotective effects of VEGF (Nicoletti et al., 2008). Therefore, the use of andiopoetin-1 can be a therapeutic strategy to suppress vascular abnormalities in the epileptic brain.

The donor and recipient cells of VEGF remain to be studied. Neurons do not express VEGFR2, which mainly plays a role in angiogenesis under normal conditions, but some studies have reported that it does play a role under abnormal conditions, such as in ischemia (Croll et al., 2004). Thus, it is proposed that cells around the NVU, including neurons, astrocytes, and endothelial cells, could express VEGFR2 after SE. The enhanced VEGF/VEGFR2 signaling in these cells may be neuroprotective in the acute phase and may contribute to angiogenesis in the chronic phase through the activation of the Src pathway and subsequent ZO-1 downregulation. Additionally, VEGF released from activated microglia in the epileptic brain may contribute to enhanced neurogenesis (Dudvarski Stankovic et al., 2016).

Overall, VEGF and VEGF signaling are potential therapeutic targets for epilepsy through the modulation of NVU structure and function. However, the donor–recipient relationships between cells that comprise the NVU should be more specifically defined in the future. It will also be important to pay attention to the opposing effects of VEGF in modulating epileptogenesis, which likely depend on the phase of epileptogenesis.

## Author Contributions

AO and RK wrote the manuscript. AO, RK, and YI discussed the manuscript.

## Funding

This work was supported in part by a Grant-in-Aid for Scientific Research (B) (17H03988 to RK) from JSPS, JST PRESTO (JPMJPR18H4 to RK), and JST ERATO (JPMJER1801 to YI).

## Conflict of Interest

The authors declare that the research was conducted in the absence of any commercial or financial relationships that could be construed as a potential conflict of interest.
